# Mechanism of network pharmacology of Erzhi Pill and Erxian Decoction in treating climacteric syndrome with “treating the same disease with different methods”: A review

**DOI:** 10.1097/MD.0000000000038440

**Published:** 2024-06-07

**Authors:** Lin-Lin Chen, Guang Zhu, Ji-Ping Xie

**Affiliations:** a Department of Gynaecology, Tongde Hospital of Zhejiang Province, Hangzhou, China.

**Keywords:** Erxian Decoction, Erzhi Pill, mechanism prediction, network pharmacology, same disease with different treatments

## Abstract

Network pharmacology and molecular docking methods were used in the present study to clarify the molecular mechanism of two traditional Chinese medicine prescriptions of climacteric syndrome. Based on oral availability and drug similarity, the main active components of Erzhi Pill and Erxian Decoction were screened through the platform of traditional Chinese medicine system pharmacology. The target database of climacteric syndrome was established by using GENECARD, OMIM, PharmGKB, Targets and Drugbank. The “component – target” network diagram was constructed using Cytoscape software (version 3.8.2). Topology analysis, module analysis, and GO and KEGG enrichment analyses were used to explore the core target and action pathway of Erzhi Pill-Erxian Decoction for treating climacteric syndrome of same disease with different treatments. There were 16 active components and 103 corresponding targets found in Erzhi Pill; 69 active components and 121 corresponding targets were found in Erxian Decoction; and 100 potential targets were found in Erzhi Pill and Erxian Decoction. Through network analysis, topology and module analysis, TP53, AKT1, Jun, ESR1, IL1B, CASP3, MMP9, PTGS2, HIF1A, MYC and EGFR could be considered as potential targets of the 2 prescriptions for alleviating climacteric syndrome. The effects of Erzhi pill and Erxian Decoction on climacteric syndrome are mainly in the pathway of lipid and atherosclerosis, AGE-RAGE signaling pathway and PI3K-Akt signaling pathway in diabetic complications. The active components in Erzhi Pill - Erxian Decoction, such as quercetin, show considerable potential as a candidate drug for the treatment of climacteric syndrome.

## 1. Introduction

Menopausal syndrome refers to a group of syndromes before and after menopause (45–55 years old), due to the fluctuation or decrease of sex hormones caused by the decline of ovarian function, resulting in vasodilatation and autonomic dysfunction and psychoneurological anomalies, with menstrual dysfunctions, hot flashes, sweating, insomnia, emotional anomalies, and bone, joint, and muscular pains as the main manifestations, which is also known as the “perimenopausal syndrome.” It is also known as “perimenopausal syndrome,” and in Chinese medicine, it is called “all the symptoms before and after menopause” and “all the symptoms before and after menstrual break.” Its incidence and severity are affected by various factors such as geography, race and individual differences.^[1]^ Menopause is a physiological event in a woman’s life and natural part of the aging process. The transition from full ovarian function in the premenopausal period to a complete lack of ovarian estrogen synthesis in the postmenopausal period is a natural process but can greatly influence daily life in women and lead them to seek medical help. For some women, these symptoms may have a significant negative impact on quality of life.^[2]^ Data statistics: in 2010, there were about 160 million climacteric women in China, and more than 120 million women were troubled by climacteric syndrome (CS) every year, which seriously affected the quality of life and work. Improving the quality of life of menopausal women has been listed as one of the 3 major health issues in the 21st century.^[3]^ At present, the treatment of CS by western medicine is still mainly hormone therapy, supplemented by symptomatic treatment.^[4,5]^ However, there are some adverse effects and risks associated with hormone therapy, such as increasing the patient’s risk of endometrial cancer, breast cancer, abnormal uterine bleeding, and cardiovascular diseases.^[6]^ There are widespread fears and misunderstandings about hormone preparations in patients, which reduce the compliance of hormone therapy.

There are notable methods of traditional Chinese medicine (TCM) in the treatment of CS. TCM can achieve a good long-term curative effect after dialectical treatment, with minimal side effects. Such treatment has been accepted and recognized by people, and has distinct advantages over modern medicine for the treatment of CS.

Erzhi Pill consists of Ligustrum lucidum and Eclipta, modern studies have found that Erzhi Pill has a wide range of pharmacological activities in perimenopausal syndrome, hepatoprotection, osteoporosis, immune system, anti-aging, and neurological disorders.^[7]^ In gynecological diseases, Erzhi Pill can relieve a series of autonomic nervous system dysfunction syndromes caused by fluctuation or decrease of sex hormones in ovarian function decline, including paroxysmal baking heat and sweating, irritability, palpitation and insomnia, hot flashes, red face, and emotional restlessness, etc.^[8]^ Erxian Decoction is a prescription created by Professor Zhang Bo-Na for treating diseases caused by functional decline of ovary and estrogen decrease.^[9]^ This formula is made up of Xianmao, Epimedium, Bacopa monnieri, Angelica sinensis, Zhimu, and Phellodendron Bark. The primary effects are inhibiting ovarian apoptosis, and improving the estrogen level in the body. Through modern pharmacological research, Erxian Decoction has been found to regulate reproductive endocrine from the levels of gonadotropin, hypothalamic reproductive regulatory hormone, HPOA and ovarian granulosa cells. Further, Erxian Decoction can change the level of reproductive hormone, regulate the balance between the hypothalamic – pituitary – ovarian – uterine female sexual axis in the body, promote female autonomic nerve function, increase immune improvement, and improve a series of CS caused by increased gonadotropin level and decreased estrogen level in women.^[10]^ The combination of both sides can improve the symptoms of patients with CS, enhance their quality of life, and regulate their immune function. For CS, taking into account the use of hormones in western medicine to replace clinical side effects, the balance of yin and yang can be restored through TCM and treating both the manifestation and root cause of disease, thereby improving certain ovarian functions and the level of estrogen.

**Figure 1.**
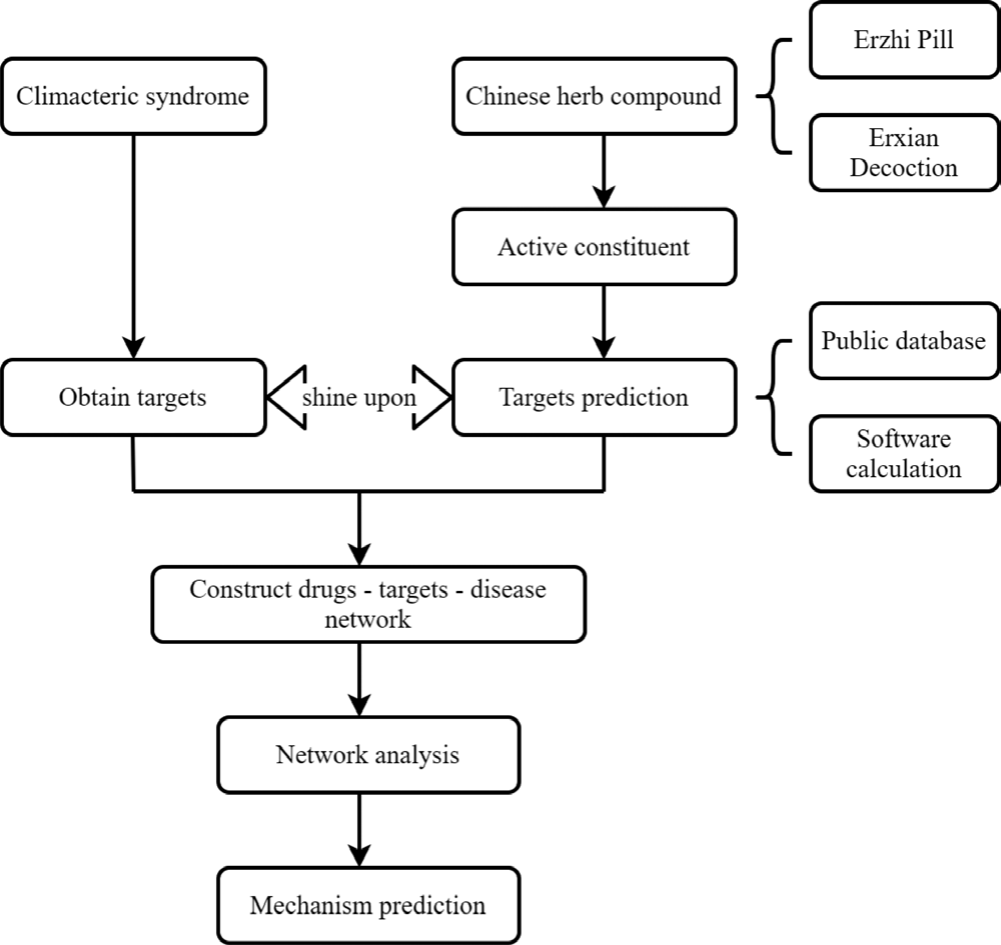
The study flow chart.

In TCM, the concept of “treating the same disease with different methods” was proposed thousands of years ago, that is, the same disease can be treated according to different people, times and places, or due to the development of the disease, different disease types and different changes of pathogenesis. Essentially, different treatment methods should be adopted according to different situations.^[11]^ Such concept has gradually become the essence of TCM treatment, and the application characteristics thereof reflect the characteristics of TCM diagnosis and treatment of diseases that emphasize the combination of syndrome differentiation, disease diagnosis and treatment.^[12]^ In the present study, the network pharmacology method was used to construct the network target and pathway combination of Erzhi pill-Erxian Decoction, the key protein for alleviating CS was found, and the action mechanism of “treating the same disease differently” for CS was revealed. The present study provides a basis for explaining the scientific connotation and pharmacological action mechanism of TCM (see Fig. 1).

## 2. Data and methods

### 2.1. Screening of CS targets

With “perimenopausal syndrome” or “menopause” as the keyword, data were searched in GenecCards Database (http://www.genecards.org/),^[13]^ Human Mendelian Genetic Database (OMIM) (http://omim.org/, Update in 2022-07-03),^[14]^ Pharmacogenomics Knowledge Database (PharmGKB) (http://www.pharmgkb.org/),^[15]^ Drugbank Database (http://www.drugbank.ca/)^[16]^ and Therapeutic Target Database (Therapeutic Targets) (http://bidd.nus.edu.sg/group/ttd/ttd.asp, TTD Vision 4.3.02).^[17]^ To obtain the data set of disease target genes, the repeated targets were removed after merging. The common targets of CS gene and Erzhi Pill-Erxian Decoction were compared and analyzed with R package, and the intersection targets of disease and drugs were displayed through a Venn diagram. The intersection targets were used as a bridge to construct the regulation network of TCM. This study did not involve human or animal subjects, and thus, no ethical approval was required. The study protocol adhered to the guidelines established by the journal.

### 2.2. Screening of active components and targets of Erzhi Pill and Erxian Decoction

Through TCMSP (https://www.tcmsp-e.com/),^[18]^ the active components and targets with 8 kinds of TCM were searched under the screening conditions of OB (oral bioavailability, OB) ≥ 30% and DL (drug likeness, DL) ≥ 0.18 in Erzhi pill-Erxian Decoction. Subsequently, the species was limited to humans through the protein database (UniProt), and then the genes corresponding to each target were corrected to obtain the standardized name abbreviation (Symbol).

### 2.3. Construction of “active ingredient-target” TCM compound regulation network of Erzhi Pill-Erxian Decoction

Drug active ingredient information, intersection target genes and CS target genes were introduced into Cytoscape 3.8 2 software,^[19]^ the regulation network of “active ingredient-target” was constructed, and the correlation between the Erzhi Pill and Erxian Decoction active ingredients and the disease genes of CS.

### 2.4. Construction of protein-protein interaction network (PPI) of common target of CS gene and Erzhi Pill-Erxian Decoction

The intersection targets obtained in 1.3 were imported into the String Database,^[20]^ the common targets of Erzhi Pill – Erxian Decoction and CS were imported into the multiple proteins function, and the organization was selected. “Homo sapiens” was selected to construct the common target PPI of Erzhi Pill – Erxian Decoction and CS, respectively. For the parameter settings, the minimum interaction score was 0.4 by default. Disrupted nodes were hidden in the network.

The obtained information was then imported into Cytoscape 3.8.2 software in the form of tsv to construct Erzhi Pill, Erxian Decoction and CS, and the common or unique active ingredients of both prescriptions for the climacteric syndrome target points network. Topological and modular analysis could be used to obtain the relationship between Erzhi Pill – Erxian Decoction targets and CS.

### 2.5. Gene ontology (GO) function enrichment analysis and Kyoto Encyclopedia of genes and genomes (KEGG) pathway analysis

The gene abbreviation (symbol) was transformed into gene ID, and then the GO (Gene ontology) enrichment and KEGG (Kyoto encyclopedia of genes and genomes) pathway enrichment of “Drug-Disease” gene were analyzed by R software. Subsequently, the relevant action pathway map was drawn.

### 2.6. Verification of molecular docking between active ingredients and target genes

The 2D structure of Erzhi Pill-Erxian Decoction for CS related target active components was obtained in the organic small molecule bioactivity database (PubChem, https://pubchem.ncbi.nlm.nih.gov/),^[21]^ and the 3D structure of the target was obtained in the protein structure database (PDB). PyMOL software was used to complete the treatment of protein receptor structure dehydrator and invalid small molecule ligand. Finally, Vina was used for molecular docking. The binding relationship and binding position between active molecules and receptor protein in Erzhi Pill-Erxian Decoction were obtained.

## 3. Results

### 3.1. Screening gene of CS

**Figure 2.**
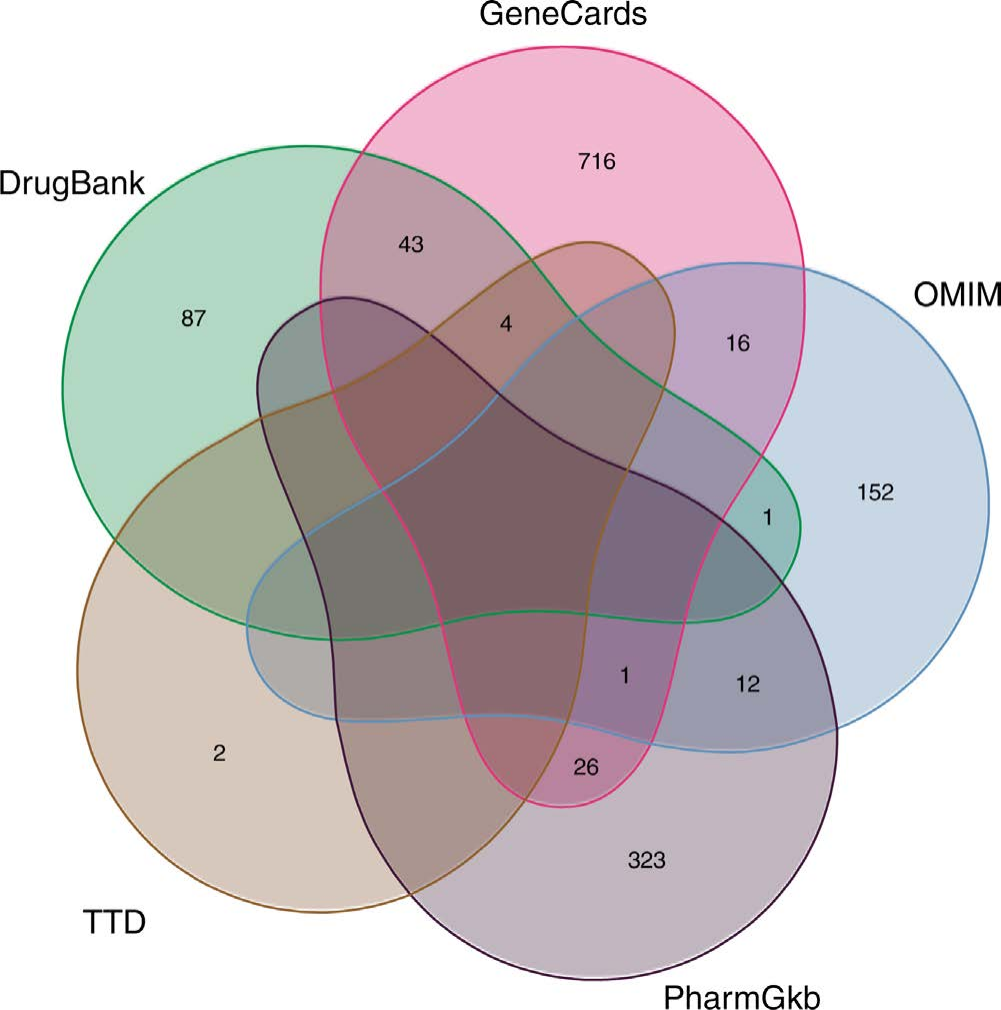
Basic information of composition and target from Erzhi Pill and Erxian Decoction.

In the databases of GeneCards, OMIM, PharmGKB, TTD, Drugbank and others were searched with “perimenopausal syndrome” or “menopause” as the keywords. The genes retrieved were filtered with relevance score > 1.0 in the GeneCards database. After searching the retrieved genes in 5 databases, a total of 1383 gene targets of CS was obtained, and then the Venn diagram was drawn (see Fig. 2).

### 3.2. Screening of effective active ingredients of Erzhi Pill – Erxian Decoction

In the TCMSP database searched by OB and DL, Erzhi Pill had 16 active ingredients, including 9 kinds in Eclipta and 9 kinds in Privet; 204 corresponding targets were found. There were 69 kinds of effective chemical components found in Erxian Decoction, including 15 kinds in Morinda officinalis, 2 kinds in Angelica sinensis, 23 kinds in Golden cypress, 6 kinds in Star grass, 22 kinds in Longspur epimedium and 12 kinds in Rhizoma anemarrhenae; 233 corresponding targets were found. The basic information of Erzhi Pill and Erxian Decoction are shown in Tables 1 and 2.

**Table 1:** Information of ingredients number and targets number from Erzhi Pill and Erxian Decoction.

Compound	Single substance drug	Number of components	Number of targets	Component-target correspondence
Erzhi Pill	Eclipta	9	86	143
	Privet	9	98	173
Erxian Decoction	Morinda officinalis	15	28	68
	Angelica sinensis	2	30	44
	Golden cypress	23	105	273
	Star grass	6	36	53
	Longspur epimedium	22	112	265
	Rhizoma anemarrhenae	12	60	108

**Table 2:** Basic information of active compounds form Erzhi Pill and Erxian Decoction.

Single substance drug	Mol ID	Molecule name	OB	DL
Eclipta	MOL001790	Linarin	39.84373106	0.70925
	MOL001689	acacetin	34.97357273	0.24082
	MOL002975	butin	69.93909366	0.2124
	MOL003378	1,3,8,9-tetrahydroxybenzofurano[3,2-c]chromen-6-one	33.93907488	0.43163
	MOL003389	3’-O-Methylorobol	57.40900238	0.2748
	MOL003398	Pratensein	39.06339634	0.27786
	MOL003402	demethylwedelolactone	72.12735394	0.42958
	MOL003404	wedelolactone	49.60209274	0.47879
	MOL000006	luteolin	36.16262934	0.24552
	MOL000098	quercetin	46.43334812	0.27525
Privet	MOL000358	beta-sitosterol	36.91390583	0.75123
	MOL000422	kaempferol	41.88224954	0.24066
	MOL004576	taxifolin	57.84156034	0.27345
	MOL005146	Lucidumoside D	48.86748631	0.70925
	MOL005147	Lucidumoside D_qt	54.4089068	0.47408
	MOL005169	(20S)-24-ene-3β,20-diol-3-acetate	40.22999698	0.81525
	MOL005190	eriodictyol	71.7926526	0.24372
	MOL005195	syringaresinol diglucoside_qt	83.1227686	0.7995
	MOL005209	Lucidusculine	30.10509486	0.74706
	MOL005211	Olitoriside	65.4545739	0.22881
	MOL005212	Olitoriside_qt	103.2309681	0.77595
	MOL000006	luteolin	36.16262934	0.24552
	MOL000098	quercetin	46.43334812	0.27525
Morinda officinalis	MOL001506	Supraene	0.42161	0.42161
	MOL002879	Diop	0.39247	0.39247
	MOL002883	Ethyl oleate (NF)	0.19061	0.19061
	MOL000358	beta-sitosterol	0.75123	0.75123
	MOL000359	sitosterol	0.7512	0.7512
	MOL006147	Alizarin-2-methylether	0.20971	0.20971
	MOL009495	2-hydroxy-1,5-dimethoxy-6-(methoxymethyl)-9,10-anthraquinone	0.37249	0.37249
	MOL009496	1,5,7-trihydroxy-6-methoxy-2-methoxymethylanthracenequinone	0.37789	0.37789
	MOL009500	1,6-dihydroxy-5-methoxy-2-(methoxymethyl)-9,10-anthraquinone	0.33917	0.33917
	MOL009503	1-hydroxy-3-methoxy-9,10-anthraquinone	0.20915	0.20915
	MOL009504	1-hydroxy-6-hydroxymethylanthracenequinone	0.2115	0.2115
	MOL009513	2-hydroxy-1,8-dimethoxy-7-methoxymethylanthracenequinone	0.37164	0.37164
	MOL009519	(2R,3S)-(+)-3′,5-Dihydroxy-4,7-dimethoxydihydroflavonol	0.33461	0.33461
	MOL009524	3beta,20(R),5-alkenyl-stigmastol	0.75074	0.75074
	MOL009525	3beta-24S(R)-butyl-5-alkenyl-cholestol	0.82221	0.82221
	MOL009537	americanin A	0.34901	0.34901
	MOL009541	Asperuloside tetraacetate	0.81584	0.81584
	MOL009551	isoprincepin	0.77375	0.77375
	MOL009558	2-hydroxyethyl 5-hydroxy-2-(2-hydroxybenzoyl)-4-(hydroxymethyl)benzoate	0.25772	0.25772
	MOL009562	Ohioensin-A	0.75842	0.75842
Angelica sinensis	MOL000358	beta-sitosterol	36.91390583	0.75123
	MOL000449	Stigmasterol	43.82985158	0.75665
Golden cypress	MOL001454	berberine	36.86124504	0.77665
	MOL001458	coptisine	30.671852	0.85647
	MOL002636	Kihadalactone A	34.20897014	0.81734
	MOL013352	Obacunone	43.28625365	0.76724
	MOL002641	Phellavin_qt	35.85997645	0.44228
	MOL002643	delta 7-stigmastenol	37.42312067	0.75103
	MOL002644	Phellopterin	40.18555771	0.27878
	MOL002651	Dehydrotanshinone II A	43.76228599	0.40019
	MOL002652	delta7-Dehydrosophoramine	54.45026528	0.25296
	MOL002656	dihydroniloticin	36.42587559	0.81454
	MOL002659	kihadanin A	31.60457831	0.70223
	MOL002660	niloticin	41.41426899	0.81833
	MOL002662	rutaecarpine	40.30045969	0.59819
	MOL002663	Skimmianin	40.13654637	0.19638
	MOL002666	Chelerythrine	34.18377333	0.77992
	MOL000449	Stigmasterol	43.82985158	0.75665
	MOL002668	Worenine	45.833181	0.86552
	MOL002670	Cavidine	35.64183046	0.80513
	MOL002671	Candletoxin A	31.81119618	0.68823
	MOL002672	Hericenone H	38.99689238	0.63395
	MOL002673	Hispidone	36.18095284	0.82983
	MOL000358	beta-sitosterol	36.91390583	0.75123
	MOL000622	Magnograndiolide	63.70888436	0.18833
	MOL000762	Palmidin A	35.35818795	0.65003
	MOL000785	palmatine	64.60111294	0.64524
	MOL000787	Fumarine	59.26250458	0.82694
	MOL000790	Isocorypalmine	35.76844006	0.59227
	MOL000098	quercetin	46.43334812	0.27525
	MOL001131	phellamurin_qt	56.59655543	0.39283
	MOL001455	(S)-Canadine	53.83441524	0.77467
	MOL001771	poriferast-5-en-3beta-ol	36.91390583	0.75034
	MOL002894	berberrubine	35.73551127	0.7269
	MOL005438	campesterol	37.57681789	0.71488
	MOL006392	dihydroniloticin	36.42587559	0.8152
	MOL006401	melianone	40.52938372	0.77799
	MOL006413	phellochin	35.41196286	0.81528
	MOL006422	thalifendine	44.41094353	0.72588
Star grass	MOL001607	ZINC03982454	36.91390583	0.75559
	MOL003578	Cycloartenol	38.68565906	0.78093
	MOL000358	beta-sitosterol	36.91390583	0.75123
	MOL004114	3,2′,4′,6′-Tetrahydroxy-4,3′-dimethoxy chalcone	52.69264509	0.28064
	MOL004125	Curculigoside B_qt	83.3623598	0.19458
	MOL004146	curculigosaponin C	39.3061041	0.18984
	MOL000449	Stigmasterol	43.82985158	0.75665
Longspur epimedium	MOL001510	24-epicampesterol	37.57681789	0.71413
	MOL003542	8-Isopentenyl-kaempferol	38.04433524	0.3948
	MOL004380	C-Homoerythrinan, 1,6-didehydro-3,15,16-trimethoxy-, (3.beta.)-	39.13992598	0.49461
	MOL004394	Anhydroicaritin-3-O-alpha-L-rhamnoside	41.5834004	0.60981
	MOL004425	Icariin	41.5834004	0.61051
	MOL000422	kaempferol	41.88224954	0.24066
	MOL001645	Linoleyl acetate	42.10076623	0.19845
	MOL004373	Anhydroicaritin	45.41193421	0.43786
	MOL004384	Yinyanghuo C	45.67199685	0.50155
	MOL000098	quercetin	46.43334812	0.27525
	MOL004391	8-(3-methylbut-2-enyl)-2-phenyl-chromone	48.54449639	0.25066
	MOL004386	Yinyanghuo E	51.63212506	0.5474
	MOL004396	1,2-bis(4-hydroxy-3-methoxyphenyl)propan-1,3-diol	52.31424958	0.22066
	MOL004382	Yinyanghuo A	56.9573795	0.76747
	MOL004388	6-hydroxy-11,12-dimethoxy-2,2-dimethyl-1,8-dioxo-2,3,4,8-tetrahydro-1H-isochromeno[3,4-h]isoquinolin-2-ium	60.64150904	0.65693
	MOL004367	olivil	62.22859563	0.40642
	MOL000622	Magnograndiolide	63.70888436	0.18833
Rhizoma anemarrhenae	MOL001677	asperglaucide	58.01629624	0.51972
	MOL003773	Mangiferolic acid	36.1592988	0.84358
	MOL000422	kaempferol	41.88224954	0.24066
	MOL004373	Anhydroicaritin	45.41193421	0.43786
	MOL004489	Anemarsaponin F_qt	60.06341209	0.78538
	MOL004492	Chrysanthemaxanthin	38.72398115	0.58352
	MOL004497	Hippeastrine	51.64996125	0.61953
	MOL004514	Timosaponin B III_qt	35.25900074	0.87013
	MOL000449	Stigmasterol	43.82985158	0.75665
	MOL004528	Icariin I	41.5834004	0.60997
	MOL004540	Anemarsaponin C_qt	35.49931721	0.86988
	MOL004542	Anemarsaponin E_qt	30.66690868	0.85657
	MOL000483	(Z)-3-(4-hydroxy-3-methoxy-phenyl)-N-[2-(4-hydroxyphenyl)ethyl]acrylamide	118.3477485	0.26399
	MOL000546	diosgenin	80.87792491	0.80979
	MOL000631	coumaroyltyramine	112.9015749	0.20234

### 3.3. Prediction of “drug target – disease gene” intersection target

**Figure 3.**
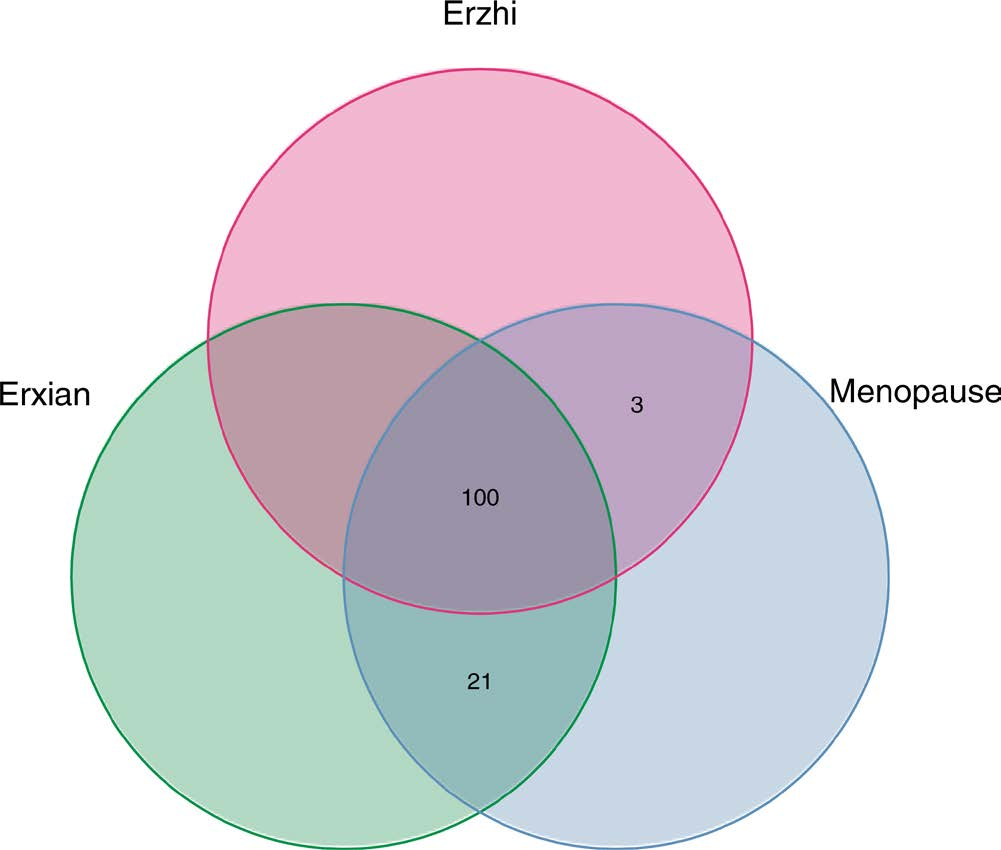
Venn diagram of 100 intersection targets from Erzhi Pill-Erxian Decoction and CS. CS = climacteric syndrome.

Via R x64 4.0 4 Venn analysis was conducted on the gene targets corresponding to the effective active components of Erzhi Pill-Erxian Decoction and the gene targets of CS diseases. Finally, 103 and 121 direct or indirect effective target gene intersections were obtained, respectively. At the same time, through analysis and calculation, the target gene mapping rates of Erzhi Pill and Erxian Decoction for alleviating CS were 55.98% and 57.62%, respectively, indicating that both were specific. The common and unique targets of Erzhi Pill-Erxian Decoction were then sorted, the Venn diagram of common targets of Erzhi Pill-Erxian Decoction and CS was drawn (see Fig. 3), and 100 intersections were obtained. Such findings indicate that there were 100 targets related to CS, that is, the potential targets of both prescriptions for alleviating CS. The specific information of gene targets was then sorted.

### 3.4. Construction, topology and module analysis of “active ingredient target gene” regulatory network

**Figure 4.**
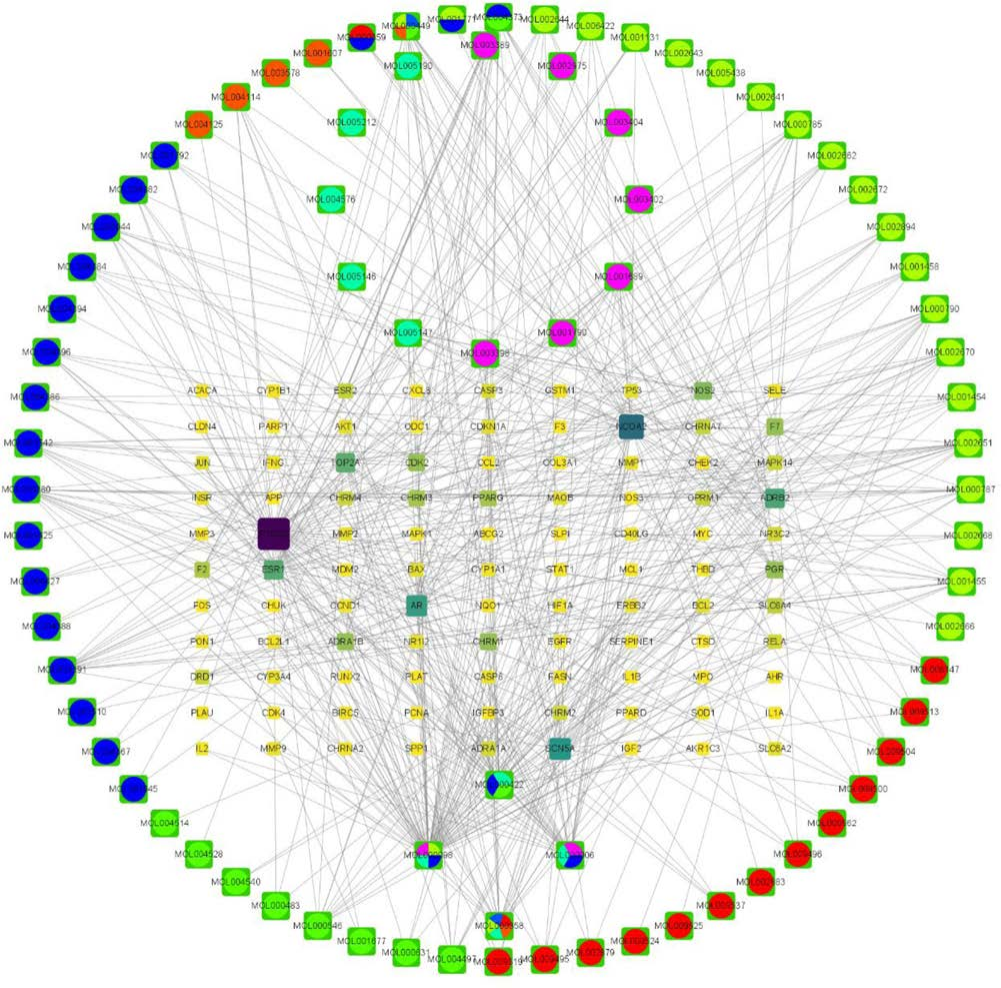
Common or unique chemical constituents of Erzhi Pill and Erxian Decoction-CS target interaction network. CS = climacteric syndrome.

The network diagram of “Erzhi Pill-Erxian Decoction drugs CS targets” was constructed by using Cytoscape software (see Fig. 4). An observation can be made that the network was composed of 124 potential target genes and 81 active components, including 12 specific active components of Erzhi Pill, with 133 targets and 244 edges, 65 specific active components of Erxian Decoction, with 121 targets and 557 edges, and 4 active components of both compounds, with 94 targets and 156 edges. Each potential target gene interacted with multiple active components, which reflected that Erzhi Pill-Erxian Decoction intervened in the treatment of CS through multiple channels, multiple components and multiple targets.

The topological analysis of the top 10 degrees was obtained according to the degree size. The topological analysis of potential targets of Erzhi Pill is shown in Table 3, the topological analysis of potential targets of Erxian Decoction is shown in Table 4, and the topological analysis of potential targets for the same disease and different treatment are shown in Table 5.

**Table 3:** Analysis of topological properties of dominant targets in Erzhi Pill (top 10 mediators).

Serial number	Gene target	Average shortest path	Degree	Betweenness	Closeness
1	AKT1	1.28712871	73	854.91437	0.77692306
2	TP53	1.36633663	71	360.90656	0.73188406
3	JUN	1.3960396	64	388.69135	0.71631205
4	IL1B	1.40594059	63	421.41483	0.7112676
5	ESR1	1.41584158	63	415.01907	0.7062937
6	CASP3	1.43564356	62	272.18457	0.69655174
7	PTGS2	1.47524752	60	197.9667	0.67785233
8	EGFR	1.44554455	60	353.6044	0.6917808
9	MMP9	1.47524752	59	195.24313	0.67785233
10	MYC	1.48514851	59	162.96056	0.67333335

**Table 4:** Analysis of topological properties of dominant targets in Erxian Decoction (top 10 mediators).

Serial number	Gene target	Average shortest path	Degree	Betweenness	Closeness
1	AKT1	1.37815126	77	1425.7736	0.7256098
2	TP53	1.54621849	72	376.1788	0.6467391
3	ESR1	1.51260504	66	695.1365	0.6611111
4	JUN	1.56302521	65	403.1015	0.63978493
5	IL1B	1.52941176	64	500.0506	0.65384614
6	EGFR	1.54621849	63	702.23474	0.6467391
7	CASP3	1.58823529	63	284.6867	0.6296296
8	PTGS2	1.61344538	62	304.1624	0.6197917
9	HIF1A	1.63865546	61	136.16862	0.61025643
10	MMP9	1.65546218	60	202.82416	0.6040609

**Table 5:** Analysis of topological properties of Erzhi Pill and Erxian Decoction (top 10 mediators).

Serial number	Gene target	Average shortest path	Degree	Betweenness	Closeness
1	AKT1	1.29591837	65	753.3588	0.766667
2	TP53	1.36734694	64	354.1645	0.724409
3	JUN	1.39795918	58	345.4877	0.713178
4	IL1B	1.40816327	58	418.4424	0.713178
5	CASP3	1.43877551	56	248.3415	0.691729
6	MMP9	1.46938776	54	167.1498	0.676471
7	PTGS2	1.47959184	54	186.4267	0.671533
8	EGFR	1.44897959	54	313.6503	0.686567
9	HIF1A	1.47959184	53	107.2104	0.666667
10	MYC	1.40816328	52	140.1623	0.661871

According to the topological analysis in the network diagram and table, the potential targets of Erzhi Pill with the top 10 degrees to alleviate CS were AKT1, TP53, JUN, IL1B, ESR1, CASP3, PTGS2, EGFR, MMP9, and MYC (see Table 3); the potential targets of Erxian Decoction for alleviating CS were AKT1, TP53, ESR1, Jun, IL1B, EGFR, CASP3, PTGS2, HIF1A, and MMP9 (see Table 4); and the potential targets of both for alleviating CS were AKT1, TP53, JUN, ESR1, IL1B, CASP3, MMP9, PTGS2, EGFR, and HIF1A (see Table 5). Based on the aforementioned information, the 11 gene targets of TP53, AKT1, JUN, ESR1, IL1B, CASP3, MMP9, PTGS2, HIF1A, MYC, and EGFR were potential targets for both prescriptions to jointly alleviate CS. An observation can be made from Figure 4 that the common active component of Erzhi pill and Erxian Decoction was MOL000098: Quercetin. There was a significant difference due to the high degree value in the network diagram, suggesting that Quercetin could be used to alleviate CS.

### 3.5. Construction of PPI network of intersection target of Erzhi Pill – Erxian Decoction and CS

**Figure 5.**
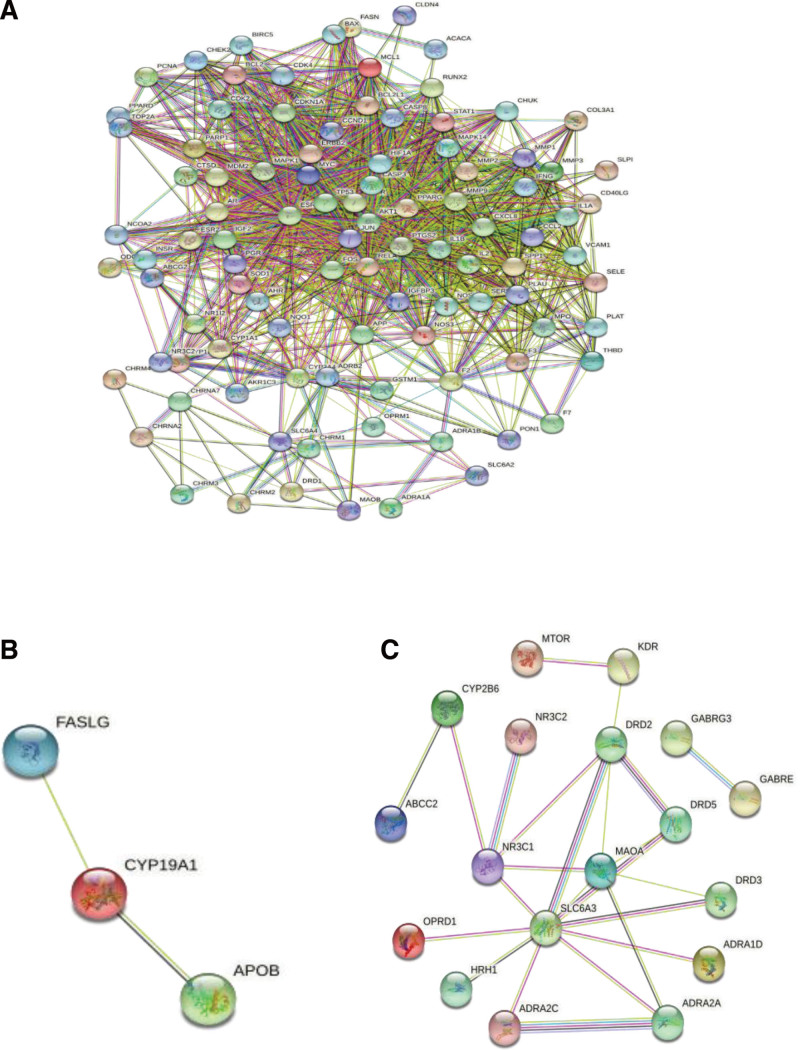
PPI network of intersection target of Erzhi Pill-Erxian Decoction and CS. (A) The network action diagram of common targets. (B) The specific network action diagram of anti-inhibition targets of Erzhi Pill. (C) The specific network action diagram of anti-inhibition targets of Erxian Decoction. CS = climacteric syndrome, PPI = protein-protein interaction network.

Using the String11.0 online tool, the common targets of the disease for both drugs was imported, in addition to the specific targets of CS for both drugs. There were 99 nodes and 1364 edges in the interaction network of the common targets of both drugs (see Fig. 5A), there were 3 nodes and 2 edges in the CS target interaction network of the specific targets of Erzhi pill (see Fig. 5B), and there were 18 nodes and 24 edges in the CS target interaction network of the specific targets of Erxian Decoction (see Fig. 5C).

The specific targets of Erzhi Pill and Erxian Decoction analyzed by String were introduced into Cytoscape, respectively. Through degree value analysis, the specific targets of Erzhi Pill were found to have higher moderate values for alleviating CS: CYP19A1, and the specific targets of Erxian Decoction were found to have higher moderate values for alleviating CS: SLC6A3, MAOA, DRD2, and NR3C1.

Cytoscape-ClusterViz was used for module analysis, and the MCODE algorithm was selected for cluster analysis of the common targets of both drugs and diseases.^[22]^ The default parameters were set with NodeScore Threshold = 0.2, and K-Core Threshold = 4. K-Core is a parameter that determines the size of the identification module, that is, the edge corresponding to the obtained module should be greater than 4. Two modules were obtained, and the numbers of genes were 40 and 15, respectively, in each module (see Table 6). R was used to analyze the genes corresponding to the module (see Table 7).

**Table 6:**
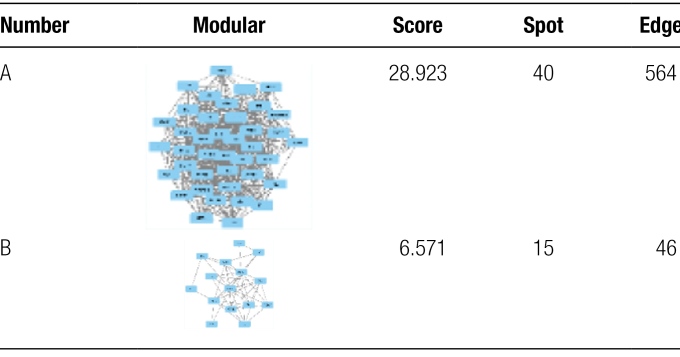
Modular analysis of common targets of Erzhi Pill and Erxian Decoction.

**Table 7:** Pathway analysis of common targets of Erzhi Pill-Erxian Decoction.

Number	Modular	KEGG function	KEGG p value
A	RELA, STAT1, MAPK1, SERPINE1, MPO, CCL2, MAPK14, CDK4, IL1A, CDK2, APP, FOS, CCND1, MMP1, AR, PGR, CASP8, BCL2L1, CASP3, MCL1, CHUK, HIF1A, ESR1, ERBB2, PLAU, MYC, IGFBP3, TP53, SPP1, AKT1, CDKN1A, EGFR, PPARG, MDM2, IL1B, NOS3, CXCL8, IL2, IFNG, JUN	hsa05220-Chronic myeloid leukemia	8.69951E-05
		hsa05215-Prostate cancer	0.000142123
		hsa04625-C-type lectin receptor signaling pathway	0.00016349
B	RUNX2, MMP9, MMP2, BAX, VCAM1, MMP3, NOS2, SELE, F3, IGF2, PARP1, PTGS2, AHR, ESR2, CD40LG	hsa04933-AGE-RAGE signaling pathway in diabetic complications	7.08334E-07
		hsa04668-TNF signaling pathway	1.24654E-06
		hsa05417-Lipid and atherosclerosis	1.34144E-06

### 3.6. Potential target GO enrichment annotation and KEGG pathway enrichment analyses

**Figure 6.**
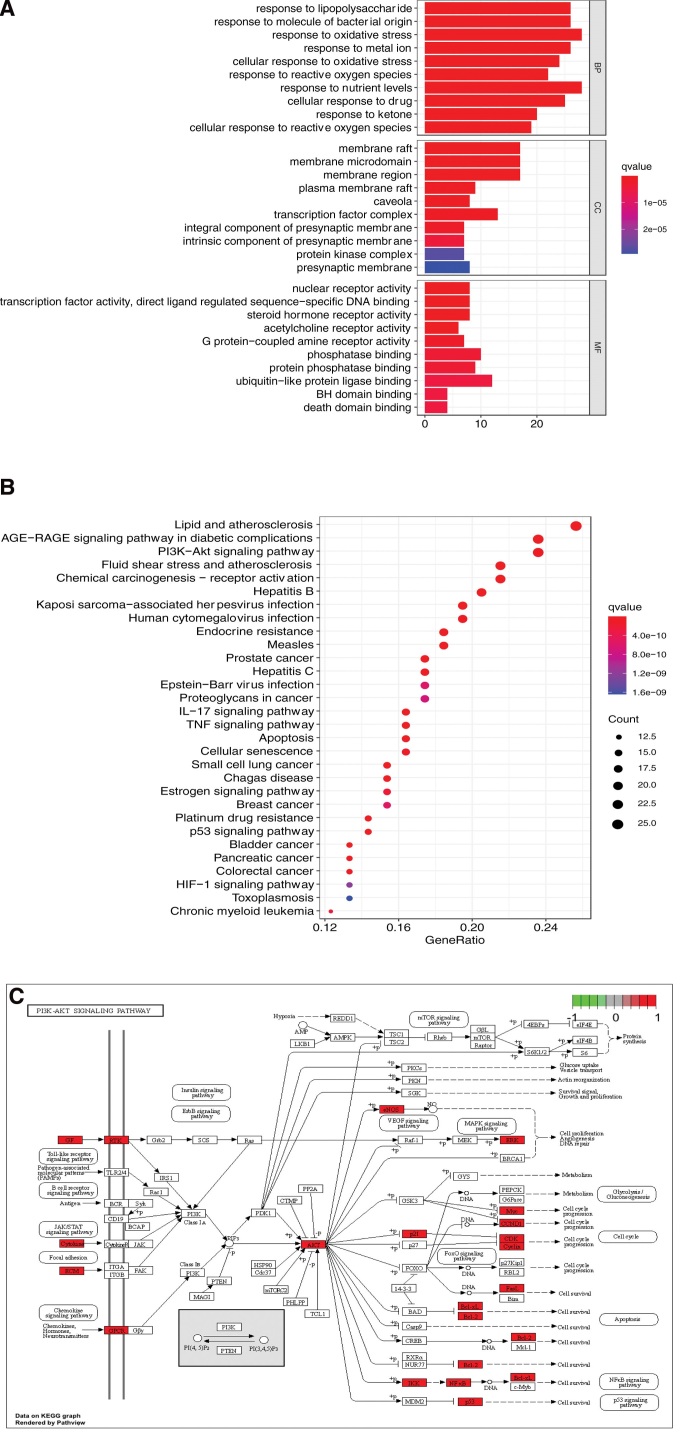
GO enrichment annotation and KEGG pathway enrichment analyses. (A) Enriched KEGG pathways of potential targets from main active ingredients of. (B) Enriched KEGG pathways of potential targets from main active ingredients of Erzhi Pill-Erxian Decoction. (C) The lipid and 4 atherosclerosis, the AGE-RAGE signaling pathway in diabetic complications and PI3K-Akt signaling pathway. GO = gene ontology, KEGG = Kyoto encyclopedia of genes and genomes.

For Erzhi Pill and Erxian Decoction, the common targets of both prescriptions would become the potential antidepressant targets of “treating the same disease with different methods” for CS. The results of GO and KEGG analysis show that in terms of enrichment results, there were 1933 of biological process, 53 of cell composition, 155 of molecular function and 151 of KEGG pathways. The results indicate that the biological processes of both prescriptions were mainly reflected in the regulation of lipopolysaccharide, molecular of bacterial origin and oxidative stress in alleviating CS. The cell composition was mainly reflected on the cell membrane. The focus of molecular functions was on nuclear receptor activity, transcription factor activity, direct ligand regulated sequence specific DNA binding and steroid hormone receptor activity (see Fig. 6A). The pathway was concentrated in the lipid and atherosclerosis, the AGE-RAGE signaling pathway in diabetic complications and PI3K-Akt signaling pathway (see Fig. 6B and C). In previous studies,^[23,24]^ the PI3K-Akt signaling pathway was found to be related to the onset of CS. The results suggest that CS might also be related to atherosclerosis and dysregulation of neurotransmitter transmission. Erxian Decoction could alleviate CS, mainly focusing on neuroactive ligand receptor interaction and dopaminergic synapse, cGMP-PKG signaling pathway and calcium signaling pathway. The specific targets of Erzhi Pill for alleviating CS are apolipoprotein B (APOB) and FASLG, which also indicates that APOB and FASLG may be different between normal and CS patients. APOB is often used to predict the severity of coronary heart disease clinically,^[25]^ and a recent study demonstrated that blood gene expression of FASLG, a Fas receptor-ligand triggering non-apoptotic inflammatory activities, is overexpressed in males and females after the age of 50,^[26]^ additional studies are encouraged to test the presence of these biomarkers in CS modality.

### 3.7. Molecular docking simulation of active ingredients and target genes

**Figure 7.**
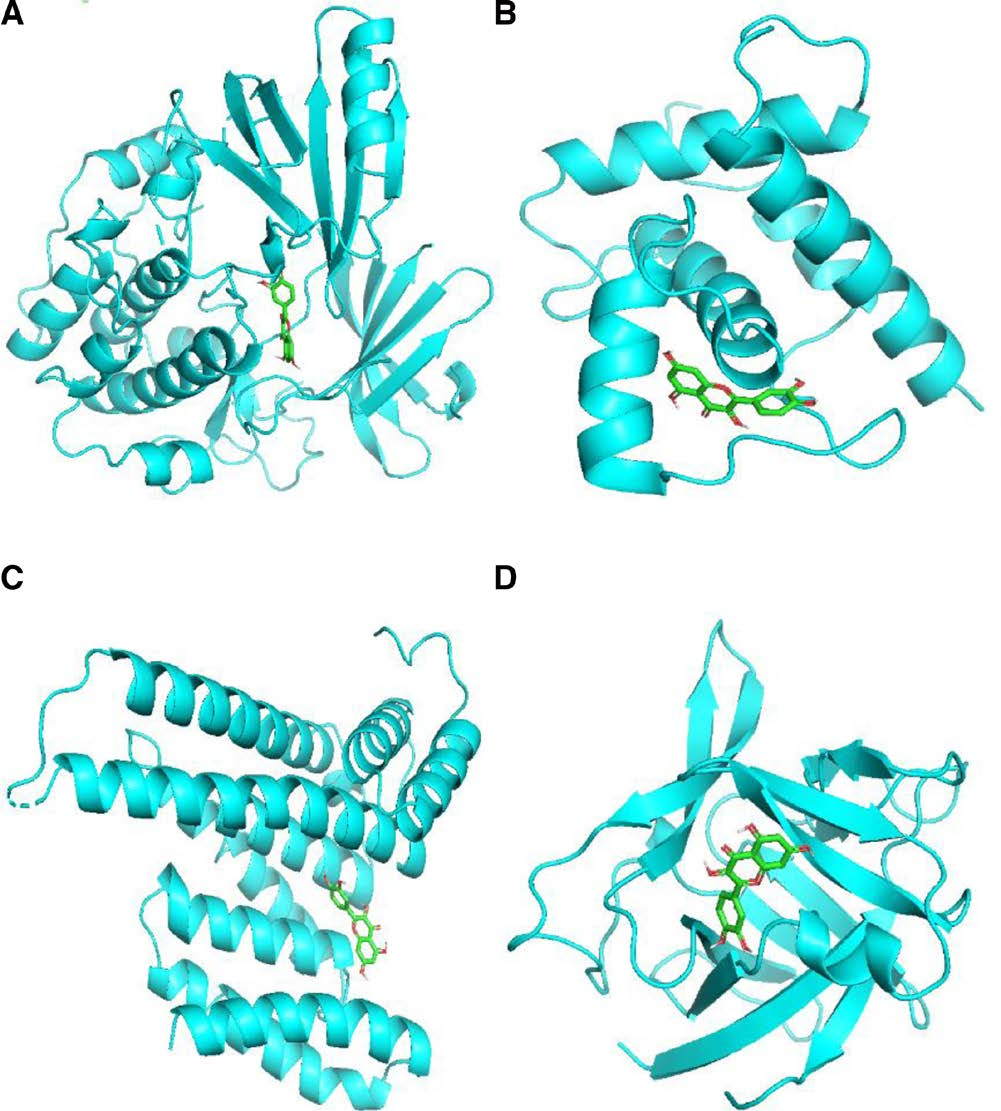
Docking mode between main active components and core targets. (A) Quercetin and AKT1 docking mode. (B) β-Sitosterol and AKT1 docking mode. (C) Luteolin and AKT1 docking mode. (D) Kaempferol and AKT1 docking mode.

The core target genes in the PPI core network were selected according to the degree value and the relevant literature on gene research of CS. The common active components and 4 molecules of Erzhi Pill and Erxian Decoction were selected through the “active component target gene” network of Erzhi Pill and Erxian Decoction. The docking results of key targets and key components were mostly lower than −5.0 kcal/mol,^[27]^ suggesting that the key components and the key targets formed a stable structure. The docking results are shown in Table 8. PyMOL software was used to visualize the docking results of the active components with the strongest binding activity of the target gene. The results are shown in Figure 7.

**Table 8:** Core targets and molecular docking results of main active components.

	AKT1	TP53	JUN	IL1B
Quercetin	−9.8	−8.3	−6.5	−5.7
β- Sitosterol	−9.6	−8.5	−7.9	−5.3
Luteolin	−9.8	−8.7	−7.7	−5.8
Kaempferol	−9.8	−8.5	−7.9	−5.4

## 4. Discussion

With the recent acceleration in the aging of the global population, the prevention and treatment of CS has become increasingly severe. The has also been a surge of interest in the research on Erzhi pill and Erxian Decoction as representative prescriptions for relieving CS. Regarding Erzhi Pill and Erxian Decoction as treatments to alleviate menopause separately, several researchers have predicted that their elaboration on the mechanism of CS often focuses on the perspective of “point” or “line” or “body.” Meanwhile, “point – line – surface – body” is rarely integrated to clarify the mechanism of treating CS. In TCM, most scholars believe that the symptoms related to menopause are only the external appearance of CS, and “oxidative stress” is the internal basic etiology of CS.^[24]^ With the help of multiple correlation analysis platforms and network pharmacology, in the present study, the effective components of Erzhi Pill-Erxian Decoction were explored, the target network between Erzhi Pill-Erxian Decoction and disease was constructed, and the interaction relationship therebetween was analyzed, so as to provide a reference for the study of the mechanism of “treating the same disease with different methods” for CS.

The prescription of TCM for the treatment of CS is established according to the specific dialectical classification, the law is established with the syndrome, and the prescription comes from the law. Based on the analysis of the pathogenesis of CS, most compounds can regulate oxidative stress, suggesting that TCM compounds can regulate oxidative stress, that is, the same disease can be treated with different methods. Although different compatibility of medicines, the dialectical treatment is different, Erzhi Pill and Erxian Decoction can alleviate CS by regulating antioxidant targets and pathways. Multiple antioxidant receptor proteins and oxidative response inhibitor proteins are located in the downstream oxidative stress inhibition pathway. The lipid and atherosclerosis signaling pathway and the AGE-RAGE signaling pathway in diabetic complications mainly involve many complex biological processes, such as cell differentiation and apoptosis, inflammatory reaction, metabolism and so on. PI3K-Akt signaling pathway can start the gene expression of downstream phase II detoxification enzymes, antioxidant proteins, ubiquitinases and proteasomes, and improve the resistance of cells against oxidative stress.^[28]^ A previous experiment of the research group using ovarian tissue culture in vitro showed that PTEN inhibitor could activate PI3K-Akt signal pathway and activate the dormant follicles in ovarian tissue for continued development.^[23]^ Quercetin has good superoxide quenching activity, dose-dependent can inhibit H2O2 induced cell death by up-regulating the level of Bcl-xL, and can also inhibit the production of TBARS induced by Pro oxidant drugs. The antioxidant activity of Quercetin may be mediated by Fenton reaction and negative regulation of NMDA receptor.^[29]^ Thus, Quercetin can be used to alleviate CS.

Erzhi Pill and Erxian Decoction may be alleviating factors in CS by regulating common targets. At present, there are a large number of reports on the mechanism of Erzhi Pill and Erxian Decoction in the treatment of CS. To investigate the antioxidant activity, Chen Wuyue et al^[30]^ measured the scavenging effect of Erzhi Pill on 2-diphenyl-2-picrylhydrazyl and hydroxyl radical in vitro. Erzhi Pill was found to have significant antioxidant activity in vitro and inhibited prednisolone induced zebrafish osteoporosis. By establishing a D-galactose induced aging cell model of Norway Rat Kidney, Liu Qinan et al^[31]^ found that Erzhi Pill can reduce the aging degree of Norway Rat Kidney cells induced by D-galactose, and the protective effect may be related to the antioxidant stress effect of Erzhi Pill. In another study,^[32]^ D-galactose was also used to induce aging in model rats, and the contents of mitochondrial respiratory chain complexes I and IV in rat brains were measured. Findings were made that the contents of mitochondrial respiratory chain complexes I and IV were significantly lower than those in the model control group in the Erzhi Pill treatment groups, which delayed the aging process of the body. Zhong Xunlong et al^[33]^ showed that Erzhi Pill can improve H2O2-induced oxidative stress damage of INS-1 pancreatic islet β cells and protect their cell functions. An assumption could be made that the mechanism is also related to inhibiting the production of ROS, increasing the activity of antioxidant enzymes and protein expression. A research group of the University of Hong Kong^[34]^ analyzed the main chemical components and pharmacological effects of Erxian Decoction, and discussed the molecular biological mechanism of the compatibility of Erxian Decoction to improve the mechanism of osteoporosis. A randomized double-blind placebo-controlled clinical trial was conducted on the efficacy of Erxian Decoction in alleviating CS, and the mechanism was studied in animal experiments. Findings were made that Erxian Decoction could regulate the secretion of estrogen, antioxidation, anti-lipid, anti-osteoporosis, reducing the metastasis of ovarian cancer cells and anti-vascular proliferation, improving the symptoms of climacteric hot flushes and night sweats, and improve the quality of life. Similarly, some scholars have conducted homologous randomized controlled trials, such as Huang et al^[35]^ found that Erzhi Pill may improve postmenopausal-related symptoms by affecting cardiovascular risk factors as HCY. Tian et al^[36]^ found that Erxian Decoction treatment of perimenopausal hyperlipidemia significantly relieved patients’ clinical symptoms and improved their sex hormone and lipid levels. Erxian Decoction could also alleviate CS through antioxidant stress. Erxian Decoction could be a significant factor in hypothalamus, adenohypophysis, gonads and other parts, regulate the function of thalamus pituitary ovary axis, promote the increase of sexual hormones secreted by its own gonads, and improve climacteric symptoms caused by the decrease of estrogen secretion. Such results are the same as the target found in network pharmacology prediction, suggesting that Erzhi Pill and Erxian Decoction can alleviate CS by regulating the same target pathway.

Erzhi Pill can also alleviate CS by regulating specific targets, and has the effect of alleviating sub-health and anti-aging. Network pharmacological studies have found that Erzhi Pill may have used specific targets to alleviate CS. For instance, Erzhi Pill can alleviate the climacteric syndrome target APOB, with the increase of APOB indicating atherosclerosis. APOB has significant predictive value in coronary heart disease and cerebral infarction, and is closely related to the occurrence of climacteric cardiovascular disease.^[37]^ Zhang Hezhen et al^[38]^ found that Erzhi Pill could improve estrogen deficiency, endothelial function and lower APOB through estrogen-like effects. Additionally, Erzhi Pill can also regulate FAS ligand FASLG. FAS/FASLG can induce apoptosis and maintain the stability of the internal environment and the balance of the body. Previous studies^[39]^ have found that the process of aging is actually a process in which the balance of the environment is broken in the body, certain cells die programmatically, and finally certain functions are damaged in the body. Compared with Erzhi Pill, Erxian Decoction has more single drugs and more specific targets for regulation. Erxian Decoction has the effects of improving blood circulation, regulating endocrine system, and resisting fatigue. The representative components of Erxian Decoction are significant factors in neuroactive ligand receptor interaction, dopaminergic synapse, cGMP-PKG signal pathway and calcium signal pathway, suggesting that Erzhi Pill and Erxian Decoction have multi-target and multi-channel characteristics.

To summarize, Erzhi Pill and Erxian Decoction are both the same and different in the treatment of CS due to their different prescriptions. Through the analysis of common targets such as AKT1, TP53, JUN, ESR1, IL1B and the biological processes and pathways where the common targets are located, the “same disease” of “treating the same disease with different methods” for CS is clarified. AKT1 is widely present in ovarian cells and plays an emphatic role in regulating follicular growth and development, inhibiting apoptosis and maintaining normal follicular cycles.^[40]^ TP53 is also associated with apoptosis or death of ovarian granulosa cells.^[41]^ JUN induces apoptosis in ovarian granulosa cells and inhibits estrogen production, leading to follicular dysplasia.^[42]^ ESR1 is widely present in the HPG axis and directly binds to estrogen.^[43]^ It has been demonstrated that reducing ovarian inflammation through anti-inflammation and decreasing IL1B expression can improve ovarian decline.^[44]^ It is hypothesized that the key targets of the 2 prescriptions for the treatment of CS mainly involve cell proliferation, apoptosis, hormonal effects, anti-inflammatory processes. APOB, CYP19A1, FASLG, ABCC2, ADRA2A, ADRA2B, and other specific targets and the biological process and pathway analysis of the specific targets preliminarily explain the dialectical connotation of “different treatment” for CS. It has been shown that higher blood low-density lipoprotein (LDL) containing apolipoprotein B (apoB) in middle age is associated with an increased risk of Sporadic Alzheimer’s Disease (SAD) in later life, which has led to the nomination of LDL as an important discriminating factor in the etiology of dementia.^[45]^ Other studies have shown that elevated CYP19A1 expression may be a marker of improved granulosa cell function, granulosa cell proliferation and prevention of ovarian hypoplasia.^[46]^ It has also been found that both men and women over 50 years of age have high expression of FASLG compared to younger people (20–30 years of age), which may induce a hyperinflammatory cascade of activation of specific immune cells.^[47]^ It has also been found that the ABCC2 gene may be involved in the regulation of bile acid transport and cholesterol metabolism, thus affecting the homeostasis of lipid metabolism.^[48]^ The gene for adrenergic receptor ADRA2A downregulated with age has a rich set of biological functions, most notably involvement in pathways associated with neurons, dendrites, synapses, and neuronal receptors and ion channels.^[49]^ Whereas brain aging may be naturally inhibited by adenosine receptor A2B (ADORA2B), a protein on the red blood cell membrane known to contribute to the release of oxygen from blood cells, thus making it available to the body.^[50]^ The aforementioned predicted targets are consistent with the pharmacological effects reported in previous literature, indicating the accuracy of the predicted targets. Further, there is a scarcity of reports on the remaining targets in the aforementioned discussion, which can provide a direction for the further study of the molecular mechanism of potential targets in alleviating CS in the future.

Through the network pharmacological analysis of “treating the same disease with different methods” of classical prescriptions for CS, as a TCM compound, there are the same and different targets in the treatment of CS. Such findings show the multi-component, multi-target and integrated regulation characteristics of the prescriptions. There are still many limitations in this study. At this stage, the understanding of the pathogenesis of CS is not comprehensive, and the preventive measures are not comprehensive. At the same time, the database and software as well as the method itself have certain limitations. There will be certain differences between the calculation results and clinical efficacy, which needs further research. In view of the above problems, the TCM compound for the treatment of clinical CS is different according to different types of CS. An explanation is also needed for the correlation mechanism between prescriptions and syndromes in combination with the pharmacodynamic substances of the compound. The mechanism of classical ancient prescriptions needs to be further studied in the treatment of CS.

## Acknowledgments

We thank Ji-Ping Xie (Tongde Hospital of Zhejiang Province), for his assistance in the design of the research strategy and Guang Zhu (Tongde Hospital of Zhejiang Province), for his assistance with some of the graphical outputs of the results. Neither received any compensation for his contribution to the study.

## Author contributions

**Formal analysis:** Guang Zhu

**Writing – original draft:** Lin-Lin Chen, Ji-Ping Xie
